# Work-Related Musculoskeletal Disorders in Physical Therapists: A Cross-Sectional Study

**DOI:** 10.3390/jcm13237425

**Published:** 2024-12-05

**Authors:** Victoria Peña-Curbelo, Alfonso Meneses-Monroy, L. Iván Mayor-Silva, Patricia Martín-Casas, Ángela Concepción Álvarez-Melcón

**Affiliations:** 1 PhD Programme in Medicine and Biomedical Sciences, Doctoral School, Universitat de Vic—Central University of Catalonia (UVic-UCC), C.Dr Junyent, 1, 08500 Vic, Spain; victoria.pena@uvic.cat; 2 Faculty of Nursing, Physiotherapy and Podiatry, Complutense University of Madrid, Plaza de Ramón y Cajal, 3, 28040 Madrid, Spain; limayors@ucm.es (L.I.M.-S.); pmcasas@ucm.es (P.M.-C.); angela.alvarez@ucm.es (Á.C.Á.-M.); 3 Health Research Institute of the San Carlos Clinical Hospital of Madrid (IdISSC), 28040 Madrid, Spain

**Keywords:** musculoskeletal disorders, physical therapists, prevalence, associated factors

## Abstract

Physical therapists, because of their care work, are susceptible to work-related musculoskeletal disorders due to their caregiving duties. These disorders have a significant economic and social impact. **Objectives:** To analyze the prevalence of work-related musculoskeletal disorders among physical therapists and their associated factors. **Methods:** A cross-sectional design study was conducted among physical therapists who were working in the Community of Madrid. An online questionnaire was used and distributed through the professional association. This questionnaire included sociodemographic and occupational variables as well as the Standardized Nordic Questionnaire, specifically designed for the analysis of musculoskeletal symptoms in an ergonomic or occupational health context. Data were analyzed using multivariate logistic regression models. **Results:** 212 questionnaires were analyzed. 98.1% reported a musculoskeletal problem in the past 12 months. The most affected regions were the neck (85.4%), upper back (59.4%), lower back (73.1%), shoulder (53.8%), and wrist and hand (63.2%). Neck disorders were associated with women (AOR = 2.41; 95%CI = 1.20–4.82); shoulder disorders with women (AOR = 7.79; 95%CI = 1.02–56.64) and older age (AOR = 1.06; 95%CI = 1.01–1.11); lower back disorders with women (AOR = 3.86; 95%CI = 1.11–13.49), a four-year bachelor’s degree (AOR = 3.56; 95%CI = 1.09–11.62), treating trauma patients (AOR = 0.13; 95%CI = 0.02–0.62), and using manual therapy (AOR = 0.34; 95%CI = 0.15–0.78). **Conclusions:** 98.1% of the physical therapists reported musculoskeletal symptoms in the past 12 months. Several variables were associated with musculoskeletal disorders: gender, level of education, age, and type of patient and treatment. Further research is needed to identify preventive measures that can reduce the high prevalence of musculoskeletal problems among physical therapists.

## 1. Introduction

Musculoskeletal disorders (MSDs) are the most common work-related health problem in the European Union (EU) [1]. In Spain, the 2023 Annual Report of the Observatory of Occupational Diseases showed that MSDs accounted for 40.24% of all reported cases among general workers, making them the most common work-related condition [2]. MSDs are defined as injuries or disorders affecting muscles, bones, tendons, nerves, ligaments, joints, and the circulatory system [1,3,4]. When these conditions are caused or aggravated by work-related activities, they are classified as work-related musculoskeletal disorders (WMSDs) [1,4].

WMSDs have a profound economic and social impact worldwide. In the EU, they are a leading cause of absenteeism and work-related disabilities, significantly reducing worker productivity and contributing to rising healthcare costs and compensation payments. Studies estimate that WMSDs account for up to 2% of gross domestic product in several European countries, primarily due to lost workdays and medical costs. In addition, WMSDs account for approximately one-third of disability benefits in the EU, underscoring the significant burden these conditions impose not only on individuals but also on society and the economy as a whole [1,5].

In general, these conditions do not have a single cause, but their etiology is multifactorial, often resulting from a combination of several risk factors [6]. Several groups of risk factors have been identified, including physical and mechanical factors, organizational and psychosocial factors, and individual and personal factors, which may contribute to the development of WMSDs [4,6].

Healthcare professionals are recognized as a high-risk group for WMSDs due to the physical demands of their roles, with prevalence rates exceeding 75% in most cases [1,2]. Physical therapists are particularly affected, with prevalence rates consistently exceeding 85% [3,4]. Other professions, such as occupational therapists (77.8%) and nurses (80.0%), are also significantly affected [3]. The prevalence of these disorders was further exacerbated during the pandemic, as psychosocial risk factors, including stress, emotional demands, and lack of organizational support, compounded the physical strain experienced by healthcare workers [5].

Previous studies show a high prevalence of affected individuals and suggest an increasing trend in the annual incidence of these diseases among physical therapists [7]. The regions most affected by these disorders in physical therapists are the lower back [8,9,10,11,12,13], the neck [8,9,10,11,12,13], the upper limbs, especially the shoulders [8,10,12,13], and the hands and wrists [8,9,11,13]. The most commonly reported types of injuries are muscle strains, intervertebral disk involvement, and sprains [11]. Some specific diagnoses are also identified, such as bursitis, thoracic outlet syndrome, sciatica, herniated disk, and torn meniscus [14].

The risk factors for these WMSDs in physical therapists are divided into four areas. The first relates to specific activities, such as transferring heavy patients, sudden movements, or different treatment techniques. The second relates to postural factors, including performing treatments in awkward postures or holding positions. The third relates to workload, referring to the frequency and repetition of treatments and the management of treatment with patients, including breaks [15,16]. The fourth relates to personal factors such as age, body mass index (BMI), and professional experience [13].

Several studies of WMSDs among physical therapists have examined risk factors by body region [13,17,18,19]. Neck complaints have been reported to be associated with the use of manual therapy [18] and working in the public sector [19], and with therapeutic exercise as a protective factor [19]. Shoulder complaints have been associated with female gender [17], years of experience [17,19], use of mobilizations [18], and number of patients per week [19]. Hand, wrist, and thumb complaints were associated with the female gender [17], hours worked per week [17], use of mobilizations [13,18], and working in the private sector [13]. On the other hand, lower back complaints are associated with age [13,17], years of experience [17,19], weekly working hours [19], the use of manual therapy [18], and mobilizations [13].

In addition, WMSDs lead to significant changes in the work habits of physical therapists. Many professionals adjust their practice by reducing manual therapy, changing positions frequently, or adjusting their schedules, such as limiting patient loads or taking more breaks. These disorders can also result in work interruptions, leading some physical therapists to change jobs or even consider a career change [11,13,20].

Given the multifactorial nature of the development of WMSDs, it is necessary to identify the relevant risk factors for each population group, in this case physical therapists, in order to implement effective strategies to minimize or eliminate the risks that influence the development of WMSDs [21].

Therefore, the main objective of the research was to determine the prevalence of WMSDs among physical therapists in the Community of Madrid. Furthermore, to observe which anatomical region is more susceptible to these disorders and to determine whether there is a relationship between sociodemographic and occupational variables and the presence of these disorders.

## 2. Materials and Methods

A cross-sectional study was conducted among physical therapists in the Community of Madrid. Ethical approval was obtained from the Research Ethics Committee of the Hospital Clínico San Carlos, in accordance with the ethical principles laid down in SAS Regulation 3470/2009 and the World Medical Association’s Declaration of Helsinki on the ethical principles for medical research involving human subjects and its subsequent amendments [22]. To ensure comprehensive reporting of the results of this cross-sectional study, the Strengthening the Reporting of Observational Studies in Epidemiology (STROBE) guidelines were followed [23].

### 2.1. Participants

Eligible participants for this study were active physical therapists in the Community of Madrid, who had more than one year of professional experience. The principal investigator identified and approached potential participants through professional networks and through the Professional Association of Physical therapists of the Community of Madrid.

Physical therapists were excluded if they were inactive at the time of the study, had less than one year of professional experience, or did not provide consent to participate in the study.

### 2.2. Performance Measures

Data were collected through an online questionnaire created through Google Forms and distributed through the institutional social networks of the Professional Association of Physical Therapists of the Community of Madrid and other personal social media platforms of the researchers. The questionnaire was also distributed through the institutional social media accounts of the association, as well as the personal social media platforms of the researchers.

The questionnaire was divided into two main sections. The first section included self-reported information on sociodemographic and professional variables. The sociodemographic variables collected were gender, age, weight, height, education level, and weekly time spent on physical activity. The occupational variables included sector (public or private), type of employment, years of professional experience, time in the current position, weekly hours worked, shift type, primary treatment method, primary patient type, average time spent per patient, weekly patient volume, and whether participants treated multiple patients, simultaneously.

To assess musculoskeletal symptoms, the questionnaire included the general section of the Standardized Nordic Questionnaire, a validated self-administered tool with 27 dichotomous (yes/no) questions. This section assessed musculoskeletal symptoms experienced in the past 12 months, the past 7 days, and their impact on regular activities in the past year. It covered nine anatomical regions: neck, shoulders, elbows, wrists/hands, upper back, lower back, hips/thighs, knees, and ankles/feet. A posterior anatomical diagram was included to assist participants in accurately identifying these regions [24].

The Spanish version of the Nordic Musculoskeletal Questionnaire (NMQ) has been validated in several studies in occupational health contexts, confirming its utility in assessing WMSDs in Spanish-speaking populations. In a prominent study of nursing assistants in Spain, the NMQ was translated, culturally adapted, and validated, demonstrating robust psychometric properties, including high internal consistency (ω = 0.81) and strong test–retest reliability (ICC = 0.95) [25].

### 2.3. Sample Size and Statistical Analysis

The sample size was calculated using the GRANMO online tool version 12.7 (https://www.datarus.eu/aplicaciones/granmo/, accessed on 18 October 2020). The study required a sample of 195 persons considering that the number of physical therapists registered in the Professional Association of Physical therapists of the Community of Madrid was 11,360 and that the percentage of the population in the last 12 months was expected to be around 85%, according to the literature consulted [15,26]. This is considered sufficient to estimate, with a confidence level of 95% and a precision of +/− 5 percentage units, the percentage of necessary referrals, which was predicted to be 1%.

Data were initially collected using Microsoft Excel 2016. After data collection, statistical analyses were performed using 4.1.1 of the R statistical software.

To summarize quantitative variables, we use the mean and standard deviation if they follow a normal distribution (as determined by the Kolmogorov–Smirnov test), and the median and interquartile range if they do not follow a normal distribution. Qualitative variables were summarized using frequencies and percentages.

Prior to analysis, variables were categorized according to established criteria: weekly time spent in physical activity followed WHO guidelines [27], with the cut-off point set at two and a half hours, the minimum recommended for weekly aerobic activity. Patient volume per week followed the approach of Ezzatvar et al. [19], with three intervals: ‘<30’, ‘30–50’, and ‘>50’ patients per week. The categorization of time in current position and weekly working hours used the thresholds of Cabezas et al. [26], creating the groups ‘<5 years’, ‘5–20 years’, and ‘>20 years’ for time in the current position, and ‘≤35 h’ and ‘>35 h’ for weekly working hours. To assess the prevalence of WMSDs, participants were considered positive if they reported at least one symptom in any anatomical region. Prevalence was further examined by anatomical region.

A multivariate logistic regression model was used to explore factors associated with the presence of WMSDs in different anatomical regions, assessing symptoms that affected work performance during the past 12 months and during the past 7 days. Several similar studies have reported such an association in each body region separately [13,17,18,19]. In this case, in addition, models are additionally reported for the most frequently affected body regions, as in the study by Khairy et al. [17].

For the analysis, the dependent variable WMSDs was coded as a categorical variable (yes = 1 and no = 0 responses), where yes indicates the presence of WMSDs and no indicates the absence of WMSDs in each anatomical region. Each independent variable (16 in total) was first analyzed by simple binary logistic regression by anatomical region. All variables associated at a threshold of *p* < 0.25 were retained for an initial model, a cut-off of 0.25 is supported by the literature [28,29]. Both forward and backward stepwise selection procedures were used, with the final model selected based on the Akaike Information Criterion (AIC). Selection by AIC prioritizes models that balance fit and simplicity, favoring lower AIC values [30].

Data are presented as odds ratios (OR), adjusted odds ratios (AOR), and their respective 95% confidence intervals (CI). Multicollinearity was assessed using Variance Inflation Factors (VIF), with values below 5 indicating acceptable levels of multicollinearity. The Hosmer–Lemeshow test was used to confirm the goodness of fit of the models. The Hosmer–Lemeshow GOF test is the most commonly used test for logistic regression models [31]. The level of statistical significance considered in all cases was *p* < 0.05.

## 3. Results

A total of 244 questionnaires were collected, after applying the exclusion criteria, 31 responses were because physical therapists had less than one year of work experience and 1 response was discarded because the participant did not give permission to use the data collected in the survey, leaving 212 questionnaires to be analyzed.

### 3.1. Sociodemographic and Occupational Characteristics

The sample was predominantly female (75.0%) and of postgraduate academic level (46.2%). The median age was 34.5 (14) years, and the median BMI was 19.5 (4.3) kg/m^2^. Most of the physical therapists (67.0%) spent at least 2.5 h weekly in physical activity. With regard to the employment variables, the physical therapists mainly worked in the private sector (72.6%), on contract (67.9%), in split shifts (46.2%), and more than 35 h per week (52.4%). In total, 38.7% of the physical therapists had more than 15 years of work experience, while 56.1% of the physical therapists had less than 5 years in their current job (Table 1).

### 3.2. Musculoskeletal Disorders

In terms of WMSDs, a total of 208 physical therapists (98.1%) reported suffering from a musculoskeletal problem in the past 12 months. Of the complaints suffered in the last 12 months, 89 participants (42%) were prevented from performing their usual tasks. The prevalence in the last 7 days was 79.7%. Gender differences were observed, with 75.5% of women reporting MSDs in the past 12 months compared to 24.5% of men. In terms of educational background, 46.2% of those reporting musculoskeletal problems in the past 12 months had a postgraduate education, 33.7% had a bachelor’s degree (3 years), and 20.2% had a bachelor’s degree (4 years). Furthermore, physical therapists who exercised more than 2.5 h per week reported fewer impairments (62.9%) compared to those who exercised less (37.1%) (Table 2).

#### 3.2.1. Neck

**Figure 1:**
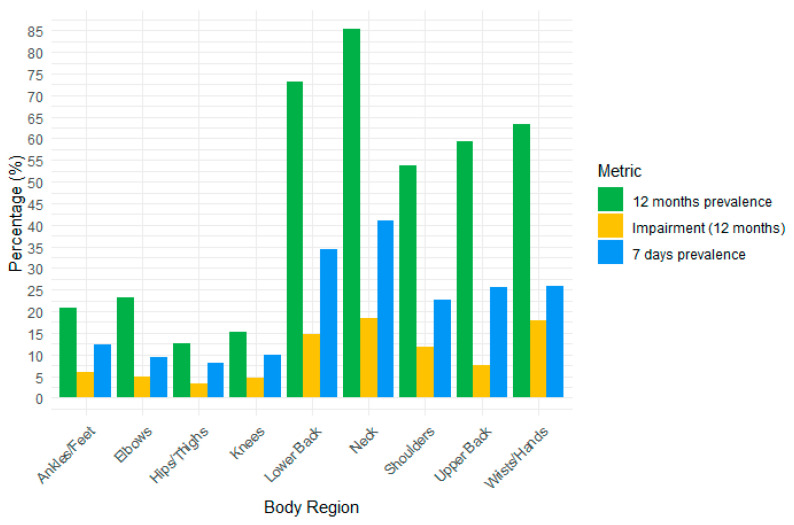
Prevalence of WMSDs by body region.

The prevalence in the past 12 months was 85.4%, for disability or impairment 18.4%, and in the last 7 days 41% (Figure 1). In the final multivariate model, working more than 35 h per week increased the risk of neck disorders in the past year (AOR = 2.46; 95%CI = 1.1–5.51) (Table 3). For disabling conditions, physical therapists with a 4-year bachelor’s degree also showed an increased risk compared to those with a 3-year degree also showed an increased risk (AOR = 4.87; 95%CI = 1.43–16.54) (Table 4). Finally, in the last 7 days, women (AOR = 2.41; 95%CI = 1.2–4.82) and those who mainly used manual therapy had a higher risk of neck disorders (AOR = 1.96; 95%CI = 1.0–3.84) (Table 5).

#### 3.2.2. Shoulder

The prevalence in the past 12 months was 53.8%, for disability or impairment 11.8%, and in the last 7 days, 22.6% (Figure 1). In the final multivariate model for the past 12 months, treating between 30 and 50 patients per week increased the risk of shoulder disorders (AOR = 2.23; 95%CI = 1.09–4.55) (Table 3). For disorders that interfered with usual tasks, being older (AOR = 1.09; 95%CI = 1.03–1.15), holding a 4-year bachelor’s degree versus a 3-year degree (AOR = 9.26; 95%CI = 2.25–38.06), and working afternoon shifts specifically reduced the risk (AOR = 0.13; 95%CI = 0.02–0.8) (Table 4). No significant factors were identified for shoulder disorders reported in the last 7 days.

#### 3.2.3. Hands and Wrists

The prevalence in the last 12 months was 63.2%, for disability or impairment 17.9%, and in the last 7 days, 25.9% (Figure 1). In the final multivariate model for disorders that interfered with usual tasks, being female was associated with a higher risk (AOR = 3.08; 95%CI = 1.01–9.4), and treating between 30 and 50 patients per week also increased the risk (AOR = 3.06; 95%CI = 1.02–9.14) (Table 4). In the final multivariate model examining disorders in the last 7 days, treating primarily trauma patients significantly increased the risk (AOR = 9.14; 95%CI = 1.17–71.14) (Table 5).

#### 3.2.4. Upper Back

The prevalence was 59.4% in the past 12 months, for disability or impairment 7.5%, and 25.5% in the last 7 days (Figure 1). In the final multivariate model for disorders in the past 12 months, treatment of trauma patients was associated with a lower risk (AOR = 0.23; 95%CI = 0.06–0.81) (Table 3). For upper back disorders in the last 7 days, treating more than one patient at a time increased the risk (AOR = 2.56; 95%CI = 1.27–5.16), and using manual therapy as the primary type of treatment was also associated with a higher risk (AOR = 3.14; 95%CI = 1.32–7.48) (Table 5).

#### 3.2.5. Lower Back

The prevalence in the past 12 months was 73.1%, for disability or impairment disorders 14.6%, and in the last 7 days 34.4% (Figure 1). In the final multivariate model for lower back disorders in the past 12 months, having a 4-year bachelor’s degree versus a 3-year degree increased the risk (AOR = 3.56; 95%CI = 1.09–11.62), as did being self-employed versus being contracted (AOR = 3.12; 95%CI = 1.12–8.66). In addition, treating more than one patient at a time (AOR = 3.23; 95%CI = 1.16–8.95) and spending more than 30 min per patient increased the risk (AOR = 5.46; 95%CI = 1.86–16.05). Weekly physical activity of 2.5 h or more was a protective factor (AOR = 0.31; 95%CI = 0.13–0.75) (Table 3).

For lower back disorders that impaired task performance, being female was associated with a higher risk (AOR = 3.86; 95%CI = 1.11–13.49). In contrast, the use of manual therapy was protective (AOR = 0.34; 95%CI = 0.15–0.78) (Table 4).

## 4. Discussion

Among physical therapists in the Community of Madrid, 98.1% reported having experienced WMSDs in the previous 12 months. This prevalence is significantly higher than reported in other Spanish studies, such as Cabezas García et al. [26] (86.7%) and Barbas et al. [15] (85.3%). However, these results are consistent with international studies, including those conducted in Florida [32] (96%) and Iran [33] (94%).

The increased prevalence of WMSDs in past years may be related to the COVID-19 pandemic. Barros et al. [34] documented a significant increase in WMSDs among healthcare workers during the pandemic, with over 60% of nurses reporting that their WMSDs were either triggered or aggravated by their work responsibilities. This rate was significantly higher than pre-pandemic levels, highlighting the increasing impact of the pandemic on the physical demands of healthcare work. In addition, a study in South Korea found that 32.3% and 18.5% of physical therapists reported symptoms of anxiety and depression, respectively [35]. Supporting this association, Campo et al. [36] identified preexisting anxiety and depression as risk factors correlated with WMSDs in healthcare workers. Further research is needed to confirm these associations and to clarify the role of the pandemic in this increased prevalence.

The most affected anatomical regions were the neck (85.4%), lower back (73.1%), and wrists and hands (63.2%). Consistent with these findings, previous research has reported these areas as the most affected by these disorders in physical therapists [15,26], with some also including the dorsal spine [37,38] and shoulders [8,19,32] in this group.

The main findings of the present study suggest that several sociodemographic and work-related factors are associated with the presence of WMSDs in physical therapists.

Regarding gender, women were more likely to have WMSDs in the neck, lower back, and hands and wrists. In this sense, a 2016 review by Vieira et al. [7] found that women were more likely to have disorders located in the neck, upper back, lower back, and hands and wrists. Subsequent studies have supported this association, with women having a higher prevalence of disorders in the neck [26,33], shoulders [33], wrists and hands [17,33], and upper back [33]. Some authors explain that this may be due to differences in muscle structure and volume, sex hormones, and biomechanical differences between men and women [33].

Older physical therapists are at increased risk for WMSDs, especially in the shoulder region. Rahmati et al. [18] found that physical therapists between the ages of 30 and 40 had a higher number of musculoskeletal complaints, while Bork et al. [37] concluded that those over the age of 50 had a lower risk of low back problems, likely due to a shift in roles to reduce physical strain. In addition, the study by Buczaj et al. [39] highlights that certain symptoms, including fatigue, dizziness, leg pain, headache, wrist pain, drowsiness, and numbness, were positively correlated with age, especially in the 40–49 and 50+ age groups. Buczaj et al. [39] also observed that job tenure plays a role, with individuals with longer tenure reporting higher incidences of WMSDs in various body regions. This relationship between age, job tenure, and symptom prevalence suggests a cumulative physical burden that increases over time, and highlights the need for preventive strategies tailored to older physical therapists.

Physical therapists with a four-year bachelor’s degree had a significantly higher risk of low back and shoulder disorders than those with a three-year degree. This finding suggests that curriculum length or structure may play a role in musculoskeletal health outcomes. As this factor has not been extensively investigated in similar studies, it would be valuable for future research to investigate whether the curriculum variation, including differences in practical training or physical workload, contributes to these differences. Understanding this relationship could inform curriculum design to mitigate musculoskeletal risks among physical therapist graduates.

More than two and a half hours of exercise out per week decreased the risk of low back disorders, and Ezzatvar et al. [40] concluded that performing at least 75 min of vigorous leisure-time physical activity per week was associated with lower levels of neck-shoulder WMSDs among physical therapists. The addition of physical activity, particularly strength training, may significantly improve the work capacity of physical therapists and reduce the risk of WMSDs. As shown by Calatayud et al. [41], engaging in high-intensity strength training at least three times per week is associated with optimal levels of work ability, which supports both physical resilience and mental health benefits in demanding roles, such as physical therapy [41,42].

Of the occupational variables associated with WMSDs, manual therapy increases the risk of WMSDs in the upper back and neck. In contrast, it acts as a protective factor at the lumbar level, reducing the likelihood of lower back disorders. Several studies associate manual therapy with an increased risk of WMSDs, particularly in the wrists and hands [26,33,37] and in the neck [19,33]. In addition, performing manual therapy while standing has been associated with an increased risk of osteoarthritis in the knees and finger joints later in life, likely due to the prolonged stress on these areas during therapy sessions [43]. Physical therapists often adopt prolonged positions with the trunk flexed for the development of manual therapy, and these postural factors may be associated with spinal disorders [13]; however, it is possible that physical therapists may suffer less from lower back pain, because the height of the couch can be adjusted during manual therapy and postures can be adopted that take more care of this region, but it is more difficult not to strain the cervical spine and the hands. Further studies are needed to confirm this assumption.

In our study, compared to treating neurological patients, treating trauma patients significantly increased the risk of WMSDs in the hands and wrists, while paradoxically decreasing the risk in the upper back. In addition, treating patients with conditions outside of these primary categories showed a protective effect at the lumbar level. In the study by Cromie et al. [13], the type of patient treated was found to influence the prevalence and location of WMSDs. Physical therapists working with patients requiring heavy physical handling, such as those in neurological and trauma specialties, reported a higher incidence of WMSDs, particularly in the neck, shoulders, and upper extremities. This finding is consistent with research by Liu et al. [44], who found that therapists working with musculoskeletal, neurological, and critical care patients are at increased risk for WMSDs due to the physical demands of treating these cases, which often involve frequent manipulation and repetitive stress from manual therapy techniques.

Our study identified several significant relationships between workload variables and the risk of WMSDs in different body regions among physical therapists. Specifically, treating 30–50 patients per week significantly increased the risk of WMSDs in both the shoulders and hands/wrists. In addition, treating more than one patient at a time was associated with an increased risk of WMSDs in the lower and upper back. Working more than 35 h per week was associated with an increased risk of neck disorders, while working more than 30 min per patient significantly increased the risk of lower back disorders. Workload is an important risk factor for the development of WMSDs [13,17,19]. Other studies of physical therapists have associated variables such as working more hours per week [13,17,19], seeing more patients per week [19], and working with more than one patient at a time [19] with the presence of WMSDs.

The consideration of workload modifications for physical therapists could serve as an important preventative measure against WMSDs. Implementing strategies, such as task rotation and swapping, can help to distribute physical and emotional demands more evenly among staff, thereby reducing the concentration of repetitive and monotonous tasks on individuals, which has been shown to reduce the risk of WMSDs. Incorporating scheduled breaks into the workday can significantly reduce the cumulative strain associated with high patient loads and extended treatment sessions [45]. These highlight the importance of structured workload management and ergonomic practices in clinical settings to support the physical health and sustainability of physical therapists.

In addition to the social and economic consequences [16], it must be considered that these disorders also have an impact on the physical therapist’s work performance [19,20]. To avoid aggravating their symptoms, physical therapists could modify their treatment techniques, as well as modify their activities of daily living and leisure activities [13,16]. Although in most cases, physical therapists reported that they do not limit their contact time with the patient to avoid future exposure, in some cases, physical therapists might change their specialty or leave the profession due to their physical problems [9,11,12,13].

Our findings provide a basis for developing targeted preventive interventions to address the high prevalence of WMSDs among physical therapists. Implementing strategies that address workload management, ergonomic practices, and physical conditioning can potentially reduce this occupational burden. Other studies have highlighted several effective preventive measures, such as incorporating regular task rotation to mitigate physical and mental strain from repetitive tasks, scheduled breaks to alleviate cumulative physical stress, and enhanced training in ergonomic techniques, especially in patient handling and posture, promoting regular strength training and aerobic conditioning, all of which have been shown to improve resilience to WMSDs and overall physical endurance in clinical roles, suggesting the benefit of incorporating these practices into daily routines [41,44,46]. These findings, from previous research, could serve as practical recommendations to support the physical health and longevity of physical therapists in demanding clinical settings.

The present study has several limitations that should be considered when interpreting the results. Because this was a cross-sectional study, causal relationships between the presence of WMSDs and the factors of interest could not be established. Furthermore, the study design did not allow us to determine the extent to which WMSDs are directly related to work activities or influenced by other variables, such as the natural increase in these disorders with age. In addition, the results may be biased by participants’ ability to recall the WMSDs they experienced, as the data were collected using self-report instruments that rely on subjective findings. As potential participants were invited to complete the questionnaire online, the participation rate could not be recorded, and a non-response analysis could not be performed. Despite these limitations, the findings underscore the critical need for tailored interventions in physical therapy workplaces. These interventions should include not only physical adjustments, such as ergonomic equipment and optimized workloads, but also organizational changes, including flexible schedules and support for mental issues related to WMSDs. Programs that focus on early detection and rehabilitation of WMSDs could further reduce long-term occupational effects.

Future longitudinal research should be conducted to better differentiate between work-related and non-work-related factors and to establish causal relationships, so that more effective preventive measures can be implemented to reduce the prevalence of these disorders among physical therapists.

## 5. Conclusions

Physical therapists in the Community of Madrid reported a high prevalence of work-related musculoskeletal disorders, particularly in the neck, upper back, lower back, shoulder, and hands/wrists.

Women had a higher risk of developing disorders in the neck, lower back, and hands/wrists than men. Older physical therapists were more likely to report shoulder disorders, while younger professionals were at a greater risk for lower back problems. Regarding education, physical therapists with a four-year bachelor’s degree had a significantly higher prevalence of lower back and shoulder disorders than those with a three-year degree. Treating between 30 and 50 patients per week was associated with a higher prevalence of shoulder, as well as hand and wrist disorders. Spending more than 30 min per patient increased the risk of lower back disorders, while treating multiple patients simultaneously was associated with lower and upper back disorders. Manual therapy, although protective for lower back disorders, was associated with an increased risk of WMSDs in the neck, upper back, wrists, and hands.

Considering these findings, it would be valuable to implement workload modification as a preventive tool for WMSDs in physical therapists. Strategies such as reducing the number of patients treated simultaneously, balancing treatment duration, and promoting regular physical activity, especially strength training, may help to reduce the occupational burden of WMSDs in this population.

## Figures and Tables

**Table 1:** Sociodemographic and occupational characteristics of the sample.

Variable (Unit)	Response	n	%	Median (IQR)
Gender	Men	53	25.0%	
	Women	159	75.0%	
Age (years)		212		34.5 (14)
BMI (kg/m^2^)		212		19.5 (4.3)
Education	Bachelor (3-years)	72	34.0%	
	Bachelor (4-years)	42	19.8%	
	Postgraduate education	98	46.2%	
Weekly time spent in physical activity	<2 and a half hours	70	33.0%	
	≥2 and a half hours	142	67.0%	
Sector	Private	154	72.6%	
	Public	44	20.8%	
	Public and private	14	6.6%	
Type of employment	Contract	144	67.9%	
	Self-employed	63	29.7%	
	Both	5	2.4%	
Experience	1–5 years	66	31.1%	
	6–15 years	64	30.2%	
	>15 years	82	38.7%	
Time in current position	<5 years	115	56.1%	
	5–20 years	78	38.0%	
	>20 years	12	5.9%	
Turnover	Morning shift	61	28.8%	
	Afternoon shift	53	25.0%	
	Split shift	98	46.2%	
Hours worked per week	≤35	101	47.6%	
	>35	111	52.4%	
Treat multiple patients at the same time	Yes	77	36.3%	
	No	135	63.7%	
Primary type of treatment	Physical exercise	55	25.9%	
	Manual therapy	137	64.6%	
	Other	20	9.4%	
Primary type of patients	Neurological	19	9.0%	
	Trauma	120	56.6%	
	Geriatric	31	14.6%	
	Other	42	19.8%	
Number of patients per week	<30	50	23.6%	
	30–50	93	43.9%	
	>50	69	32.5%	
Average time per patient	≤30 min	83	39.2%	
	>30 min	129	60.8%	

BMI: body mass index. IQR: interquartile range.

**Table 2:** Sociodemographic and occupational characteristics by 12-month WMSDs prevalence, impairment, and 7-day prevalence.

		12 Months Prevalence		Impairment (12 Months)		7 Days Prevalence	
Variable (unit)	Response	No n = 4	Yes n = 208	No n = 123	Yes n = 89	No n = 43	Yes n = 169
Gender	Men	2 (50.0%)	51 (24.5%)	41 (33.3%)	12 (13.5%)	15 (34.9%)	38 (22.5%)
	Women	2 (50.0%)	157 (75.5%)	82 (66.7%)	77 (86.5%)	28 (65.1%)	131 (77.5%)
Age (years)							
BMI (kg/m^2^)							
Education	Bachelor (3-years)	2 (50.0%)	70 (33.7%)	43 (35.0%)	29 (32.6%)	12 (27.9%)	60 (35.5%)
	Bachelor (4-years)	0 (0.00%)	42 (20.2%)	22 (17.9%)	20 (22.5%)	8 (18.6%)	34 (20.1%)
	Postgraduate	2 (50.0%)	96 (46.2%)	58 (47.2%)	40 (44.9%)	23 (53.5%)	75 (44.4%)
Weekly time spent in physical activity	<2 and a half hours	0 (0.00%)	70 (33.7%)	37 (30.1%)	33 (37.1%)	11 (25.6%)	59 (34.9%)
	≥2 and a half hours	4 (100%)	138 (66.3%)	86 (69.9%)	56 (62.9%)	32 (74.4%)	110 (65.1%)
Sector	Private	3 (75.0%)	151 (72.6%)	93 (75.6%)	61 (68.5%)	26 (60.5%)	128 (75.7%)
	Public	1 (25.0%)	43 (20.7%)	23 (18.7%)	21 (23.6%)	14 (32.6%)	30 (17.8%)
	Both	0 (0.00%)	14 (6.73%)	7 (5.69%)	7 (7.87%)	3 (6.98%)	11 (6.51%)
Type of employment	Contract	2 (50.0%)	142 (68.3%)	79 (64.2%)	65 (73.0%)	30 (69.8%)	114 (67.5%)
	Self-employed	2 (50.0%)	61 (29.3%)	43 (35.0%)	20 (22.5%)	10 (23.3%)	53 (31.4%)
	Both	0 (0.00%)	5 (2.40%)	1 (0.81%)	4 (4.49%)	3 (6.98%)	2 (1.18%)
Experience	1–5 years	0 (0.00%)	66 (31.7%)	37 (30.1%)	29 (32.6%)	10 (23.3%)	56 (33.1%)
	6–15 years	1 (25.0%)	63 (30.3%)	46 (37.4%)	18 (20.2%)	13 (30.2%)	51 (30.2%)
	>15 years	3 (75.0%)	79 (38.0%)	40 (32.5%)	42 (47.2%)	20 (46.5%)	62 (36.7%)
Time in current position	<5 years	1 (25.0%)	114 (56.7%)	66 (55.0%)	49 (57.6%)	23 (54.8%)	92 (56.4%)
	5–20 years	3 (75.0%)	75 (37.3%)	51 (42.5%)	27 (31.8%)	17 (40.5%)	61 (37.4%)
	>20 years	0 (0.00%)	12 (5.97%)	3 (2.50%)	9 (10.6%)	2 (4.76%)	10 (6.13%)
Turnover	Morning shift	2 (50.0%)	59 (28.4%)	34 (27.6%)	27 (30.3%)	15 (34.9%)	46 (27.2%)
	Afternoon shift	2 (50.0%)	51 (24.5%)	33 (26.8%)	20 (22.5%)	12 (27.9%)	41 (24.3%)
	Split shift	0 (0.00%)	98 (47.1%)	56 (45.5%)	42 (47.2%)	16 (37.2%)	82 (48.5%)
Hours worked per week	≤35	4 (100%)	97 (46.6%)	65 (52.8%)	36 (40.4%)	20 (46.5%)	81 (47.9%)
	>35	0 (0.00%)	111 (53.4%)	58 (47.2%)	53 (59.6%)	23 (53.5%)	88 (52.1%)
Treat multiple patients at the same time	Yes	1 (25.0%)	76 (36.5%)	42 (34.1%)	35 (39.3%)	21 (48.8%)	56 (33.1%)
	No	3 (75.0%)	132 (63.5%)	81 (65.9%)	54 (60.7%)	22 (51.2%)	113 (66.9%)
Primary type of treatment	Physical exercise	1 (25.0%)	54 (26.0%)	33 (26.8%)	22 (24.7%)	19 (44.2%)	36 (21.3%)
	Manual therapy	2 (50.0%)	135 (64.9%)	80 (65.0%)	57 (64.0%)	16 (37.2%)	121 (71.6%)
	Other	1 (25.0%)	19 (9.13%)	10 (8.13%)	10 (11.2%)	8 (18.6%)	12 (7.10%)
Primary time of patients	Neurological	0 (0.00%)	19 (9.13%)	9 (7.32%)	10 (11.2%)	2 (4.65%)	17 (10.1%)
	Trauma	3 (75.0%)	117 (56.2%)	73 (59.3%)	47 (52.8%)	18 (41.9%)	102 (60.4%)
	Geriatric	0 (0.00%)	31 (14.9%)	16 (13.0%)	15 (16.9%)	10 (23.3%)	21 (12.4%)
	Other	1 (25.0%)	41 (19.7%)	25 (20.3%)	17 (19.1%)	13 (30.2%)	29 (17.2%)
Number of patients per week	<30	0 (0.00%)	50 (24.0%)	30 (24.4%)	20 (22.5%)	7 (16.3%)	43 (25.4%)
	30–50	2 (50.0%)	91 (43.8%)	54 (43.9%)	39 (43.8%)	15 (34.9%)	78 (46.2%)
	>50	2 (50.0%)	67 (32.2%)	39 (31.7%)	30 (33.7%)	21 (48.8%)	48 (28.4%)
Average time per patient	≤30 min	1 (25.0%)	82 (39.4%)	46 (37.4%)	37 (41.6%)	21 (48.8%)	62 (36.7%)
	>30 min	3 (75.0%)	126 (60.6%)	77 (62.6%)	52 (58.4%)	22 (51.2%)	107 (63.3%)

BMI: body mass index.

**Table 3:** Multivariate final analysis of WMSDs in the past 12 months and sociodemographic and occupational variables.

Variable (Unit)	Response	OR (95%CI)	AOR (95%CI)
Neck			
Years (Age)		0.97 (0.93, 1.02)	0.97 (0.92, 1.01)
Hours worked per week	≤35	1	1
	>35	2.24 (1.02, 4.96)	2.46 (1.1, 5.51)
Shoulder			
Gender	Men	1	1
	Women	0.63 (0.34, 1.19)	0.6 (0.31, 1.13)
Number of patients per week	<30	1	1
	30–50	2.02 (1, 4.05)	2.23 (1.09, 4.55)
	>50	1.31 (0.63, 2.72)	2.06 (0.83, 5.13)
Treat multiple patients at the same time	No	1	1
	Yes	0.7 (0.4, 1.22)	0.57 (0.27, 1.18)
Upper back			
Primary time of patients	Neurological		
	Trauma	0.39 (0.12, 1.23)	0.23 (0.06, 0.81)
	Geriatric	0.32 (0.09, 1.2)	0.34 (0.09, 1.28)
	Other	0.32 (0.09, 1.14)	0.29 (0.08, 1.08)
Primary type of treatment	Physical exercise		
	Manual therapy	1.85 (0.98, 3.49)	2.66 (1.22, 5.81)
	Other	0.64 (0.23, 1.82)	0.81 (0.27, 2.41)
Lower back			
Education	Bachelor (3-years)	1	1
	Bachelor (4-years)	3.52 (1.22, 10.17)	3.56 (1.09, 11.62)
	Postgraduate education	1.21 (0.62, 2.37)	1.72 (0.75, 3.96)
Type of employment	Contract	1	1
	Self-employed	1.58 (0.81, 3.06)	3.12 (1.12, 8.66)
	Both	0.32 (0.05, 2.05)	0.19 (0.02, 2.11)
Treat multiple patients at the same time	No	1	1
	Yes	1.54 (0.79, 3)	3.23 (1.16, 8.95)
Primary type of treatment	Physical exercise	1	1
	Manual therapy	1.48 (0.73, 3.03)	1.9 (0.66, 5.47)
	Other	0.48 (0.16, 1.41)	0.43 (0.11, 1.73)
Average time per patient	≤30 min	1	1
	>30 min	1.5 (0.81, 2.81)	5.46 (1.86, 16.05)
Weekly time spent in physical activity	<2 and a half hours	1	1
	≥2 and a half hours	0.47 (0.23, 0.96)	0.31 (0.13, 0.75)
Primary time of patients	Neurological	1	1
	Trauma	0.2 (0.03, 1.6)	0.2 (0.02, 1.81)
	Geriatric	0.19 (0.02, 1.72)	0.16 (0.02, 1.63)
	Other	0.06 (0.01, 0.46)	0.06 (0.01, 0.53)

OR: odds ratio. AOR: adjusted odds ratio. CI: confidence interval.

**Table 4:** Multivariate final analysis of WMSDs that interfere with performing usual tasks and sociodemographic and occupational variables.

Variable (Unit)	Response	OR (95%CI)	AOR (95%CI)
Neck			
Gender	Men		
	Women	2.61 (0.96, 7.07)	2.44 (0.85, 6.98)
Education	Bachelor (3-years)		
	Bachelor (4-years)	2.22 (0.88, 5.61)	4.87 (1.43, 16.54)
	Postgraduate education	1.08 (0.47, 2.5)	1.85 (0.68, 5.01)
Number of patients per week	<30		
	30–50	2.14 (0.85, 5.38)	2.19 (0.78, 6.18)
	>50	0.81 (0.27, 2.39)	0.76 (0.23, 2.51)
Experience	1–5 years		
	6–15 years	0.5 (0.19, 1.35)	0.53 (0.18, 1.6)
	>15 years	1.23 (0.56, 2.72)	1.71 (0.59, 4.95)
Hours worked per week	≤35		
	>35	1.81 (0.88, 3.71)	2.08 (0.94, 4.62)
Shoulder			
Gender	Men		
	Women	9.24 (1.22, 70.08)	5.4 (0.65, 45.22)
Age (years)		1.07 (1.02, 1.12)	1.09 (1.03, 1.15)
Education	Bachelor (3-years)		
	Bachelor (4-years)	1.88 (0.65, 5.46)	9.26 (2.25, 38.06)
	Postgraduate education	0.81 (0.3, 2.21)	2.47 (0.78, 7.78)
BMI		0.84 (0.71, 0.98)	0.85 (0.71, 1.03)
Turnover	Morning shift		
	Afternoon shift	0.16 (0.03, 0.75)	0.13 (0.02, 0.8)
	Split shift	0.52 (0.21, 1.26)	0.54 (0.2, 1.51)
Lower back			
Gender	Men		
	Women	3.56 (1.04, 12.24)	3.86 (1.11, 13.49)
Primary type of treatment	Physical exercise		
	Manual therapy	0.36 (0.16, 0.81)	0.34 (0.15, 0.78)
	Other	0.33 (0.07, 1.58)	0.29 (0.06, 1.45)
Hands and wrists			
Gender	Men		
	Women	3.4 (1.14, 10.1)	3.08 (1.01, 9.4)
Time in current position	<5 years		
	5–20 years	0.55 (0.24, 1.27)	0.46 (0.2, 1.1)
	>20 years	4.23 (1.24, 14.37)	3.12 (0.88, 11.03)
Number of patients per week	<30		
	30–50	2.46 (0.86, 7.03)	3.06 (1.02, 9.14)
	>50	1.88 (0.61, 5.73)	2.22 (0.7, 7.1)

OR: odds ratio. AOR: adjusted odds ratio. CI: confidence interval.

**Table 5:** Multivariate final analysis of MSDs the last 7 days and sociodemographic and occupational variables.

Variable (Unit)	Response	OR (95%CI)	AOR (95%CI)
Neck			
Gender	Men		
	Women	2.36 (1.19, 4.69)	2.41 (1.2, 4.82)
Primary type of treatment	Physical exercise		
	Manual therapy	1.96 (1.01, 3.8)	1.96 (1, 3.84)
	Other	0.96 (0.31, 2.92)	0.9 (0.29, 2.79)
Upper back			
Treat multiple patients at the same time	No		
	Yes	1.76 (0.94, 3.3)	2.56 (1.27, 5.16)
Primary type of treatment	Physical exercise		
	Manual therapy	2.18 (0.98, 4.87)	3.14 (1.32, 7.48)
	Other	1.28 (0.35, 4.73)	1.27 (0.34, 4.77)
Hands and wrists			
Primary time of patients	Neurological		
	Trauma	8.67 (1.12, 67.29)	9.14 (1.17, 71.14)
	Geriatric	6.26 (0.72, 54.74)	6.65 (0.76, 58.37)
	Other	3.6 (0.41, 31.56)	3.81 (0.43, 33.52)
BMI		0.93 (0.84, 1.03)	0.92 (0.83, 1.02)

OR: odds ratio. AOR: adjusted odds ratio. CI: confidence interval. BMI: body mass index.

## Data Availability

The datasets generated during and analyzed during the current study are available from the corresponding author on reasonable request.
